# Clinical burden and healthcare resource utilization associated with achondroplasia: a real-world observational, retrospective cohort study

**DOI:** 10.1186/s13023-025-04072-w

**Published:** 2025-11-05

**Authors:** Pranav Abraham, Gandarvaka Miles, Natalia Petruski-Ivleva, Kalyani Hawaldar, Cemre Robinson, Kenneth I. Berger

**Affiliations:** Sanofi, Bridgewater, NJ USA

**Keywords:** Achondroplasia, Real-world, HCRU, Costs

## Abstract

**Introduction:**

Achondroplasia is the most common skeletal dysplasia associated with disproportionately short stature and is associated with high disease burden and unmet medical need. However, the clinical and economic burden of achondroplasia remains unclear. This study assessed clinical and economic burden of achondroplasia in US clinical practice.

**Methods:**

This retrospective study assessed people with ≥ 1 inpatient or ≥ 2 outpatient claims with primary diagnosis codes for achondroplasia using Optum’s deidentified Clinformatics^®^ Data Mart Database (October 1, 2015, to October 1, 2023). Index date for the achondroplasia cohort was date of first achondroplasia diagnosis code. People living with achondroplasia were matched 1:5 with nonachondroplasia controls on age, sex, index date (matched by calendar date), and observation duration. Individuals required ≥ 12 months continuous post-index health plan enrollment. Per person-year (PPY) comorbidities, healthcare resource utilization (HCRU), and costs were stratified by age (< 18 and ≥ 18 years). Outcomes were assessed in a subgroup with achondroplasia and spinal stenosis (Ach-SpS). Continuous and categorical variables were compared using *t* tests and chi-square tests, respectively. HCRU and costs were assessed using generalized linear models.

**Results:**

Overall, 626 people living with achondroplasia were matched with 3,130 nonachondroplasia controls (mean [SD] age, years: 33.2 [24.4] vs. 33.1 [24.3]; 56.2% females in both). Skeletal and extraskeletal comorbidities occurred significantly more frequently in people with achondroplasia than in nonachondroplasia controls (*P*<0.01). HCRU incidence was higher in people with achondroplasia; inpatient admissions were 0.30 vs. 0.09 PPY (pediatrics) and 0.39 vs. 0.21 PPY (adults) (both *P*<0.01). People with achondroplasia had higher total healthcare costs than nonachondroplasia controls (pediatrics: $31,388 vs. $4,164 PPY; adults: $33,360 vs. $16,887 PPY), driven mainly by inpatient admissions (pediatrics: $12,232 vs. $1,170 PPY; adults: $16,703 vs. $5,207 PPY). Incidence of outpatient visits (17.47 PPY), inpatient admissions (0.52 PPY), and total healthcare costs ($45,990) were higher in people living with Ach-SpS than in the overall achondroplasia cohort.

**Conclusion:**

Achondroplasia imposes substantial clinical and economic burden in people across age groups in US clinical practice; this burden is elevated in people living with Ach-SpS. These data emphasize the unmet medical need for more effective disease-modifying therapies for achondroplasia.

**Clinical trial number:**

Not applicable.

**Supplementary Information:**

The online version contains supplementary material available at 10.1186/s13023-025-04072-w.

## Background

Achondroplasia is a rare genetic disease caused by a recurrent gain-of-function mutation of the fibroblast growth factor receptor 3 (*FGFR3*) gene [1], with a global prevalence of approximately 250,000 people [2]. It is the most common form of skeletal dysplasia [3] associated with disproportionately short stature. Achondroplasia is also associated with an increased risk of premature death [4, 5], and the life expectancy of people living with the disorder is approximately 10 years less than the general population [1]. Most clinical manifestations of achondroplasia present at birth or in early childhood; the most frequent complications include spinal stenosis, macrocephaly, midface hypoplasia, depressed nasal bridge, and a relatively small chest [1, 6, 7].

People living with achondroplasia require lifetime care by a multidisciplinary team [1, 6]. Treatment options for achondroplasia are limited; currently, the only US Food and Drug Administration (FDA)-approved pharmacological intervention is vosoritide, a C-type natriuretic peptide analogue that binds to natriuretic peptide receptor B, thus reducing the activity of *FGFR3* and allowing for bone growth. Vosoritide was approved in 2021 for use in children aged ≥ 5 years with achondroplasia and open epiphyses [8, 9]. In October 2023, this FDA approval was subsequently extended for use in children of all ages [9]. However, despite the availability of vosoritide, there is continued need for surgical interventions which aim to improve specific complications (e.g., lengthening of lower limbs or spinal decompression and fusion) [10]. These surgical procedures are associated with high treatment burden, severe complications, and are performed only on long bones (e.g., the femur or tibia) thus do not help complications relating to other bone types [7, 11]. Accordingly, the management and treatment of achondroplasia is associated with substantial clinical and economic burden and reduced quality of life in people and their families [1, 6].

With the paucity of effective interventions and management pathways, there is a high unmet medical need in people living with achondroplasia, and the clinical burden and economic impact of the disease have not been quantified. This study aims to assess the clinical, healthcare resource utilization (HCRU), and healthcare cost burden in people living with achondroplasia compared with people without achondroplasia in US clinical practice.

## Methods

### Study design and data source

This longitudinal retrospective cohort study used data from Optum’s deidentified Clinformatics^®^ Data Mart (CDM) Database (an administrative US claims database) between October 1, 2015, and October 1, 2023. CDM includes data from more than 180 million patients, including data on demographics (sex, age, dates of eligibility, health plan type), diagnoses (*International Classification of Diseases*,* Ninth and Tenth Revisions [ICD-9* and *ICD-10]* codes) and on procedures performed during outpatient visits or inpatient stays, including outpatient prescription fill records. Institutional review board approval was not required, because all data were deidentified prior to acquisition.

### Patient population

Patients in the CDM database who met the following criteria were included in the achondroplasia cohort: ≥1 claim with a primary diagnosis code for achondroplasia (*ICD-10* diagnosis code: Q77.4) during an inpatient stay or ≥ 2 claims with an achondroplasia diagnosis code on ≥ 2 distinct days in any noninpatient setting; index date was the date of first achondroplasia *ICD-10* code during the study period. Each patient in the achondroplasia cohort was then matched to 5 people without achondroplasia based on age at index (exact year matching for people aged < 18 years; 5-year age groups for people aged ≥ 18 years), sex, index date, and length of observation period. A 1:5 fixed matching ratio was used as it represented the highest ratio achievable with this dataset while preserving the largest sample of people living with achondroplasia. After index, people were required to have ≥ 12 months of continuous healthcare plan enrollment (with no healthcare coverage gaps permitted). People in the matched, nonachondroplasia control cohort were required to have a medical claim within the calendar month/year corresponding to the index date of the achondroplasia case and to have subsequent continuous healthcare plan enrollment ending between 0 and 60 days after that of the matched achondroplasia case. This approach ensured that for each nonachondroplasia control, the index date and the observation period corresponded to those of the achondroplasia case.

### Study variables

This study reported patient demographics and clinical characteristics captured at index. Comorbidities (including Charlson Comorbidity Index [CCI] scores and achondroplasia-related conditions) were captured over all available (variable length) follow-up. The total CCI score consists of a sum of the weights of 19 items corresponding to different comorbid conditions; a higher score indicates more severe comorbid conditions and a greater mortality risk [12]. Annualized all-cause HCRU and all-cause healthcare costs were assessed over all available follow-up; all outcomes were stratified by age (pediatrics aged < 18 years and adults aged ≥ 18 years). All-cause HCRU included outpatient visits, emergency room (ER) visits, inpatient admissions, long-term care (LTC), pharmacy fills, and other visits (complementary medicine services, physical medicine and rehabilitation procedures, physiotherapy, occupational therapy, chiropractic/osteopathic procedures, acupuncture, speech therapy, and medical imaging); HCRU categories were defined by place of service codes. Healthcare costs included medical service costs relating to HCRU (outpatient, ER, inpatient, LTC, and other) and pharmacy drug costs (estimated overall from the total cost of pharmacy claims). Medical service costs for each HCRU category were calculated by adding the costs for all claims associated with the episodes of care in that HCRU category; total healthcare costs were calculated by summing the medical service costs and pharmacy drug costs.

A subgroup analysis was performed assessing people living with achondroplasia of any age with ≥ 1 claim with diagnosis for achondroplasia including diagnosis codes for spinal stenosis (Ach-SpS; *ICD-10*: M4800-M4808, M48.061). Since spinal stenosis is a highly prevalent complication in achondroplasia, the purpose of this subanalysis was to assess the additional clinical and HCRU burden in this subpopulation. A sensitivity analysis assessed outcomes during a fixed (12-month) follow-up.

### Statistical methods

Counts and proportions (categorical variables) were reported for patient demographics, comorbidities, and all-cause HCRU, and comparative analyses between the achondroplasia and nonachondroplasia control cohorts were performed via chi-square tests. Means and SD (continuous variables) were reported for patient demographics and comorbidities and compared between cohorts via *t* tests. All-cause HCRU incidence rates (per person-year [PPY]) and incidence rate ratios (IRRs) comparing achondroplasia and nonachondroplasia cohorts were calculated; associated 95% CIs and *P* values were derived using unadjusted generalized linear models with a log link and Poisson distribution. Healthcare cost differences were estimated using 2-part models, which are based on a logistic regression model to calculate unadjusted mean differences and associated CIs and an unadjusted generalized linear model with gamma distribution and log link (or identity link, if a better model fit) and a robust variance estimator to estimate nonzero mean differences with bootstrapped CIs. Estimates from both models were combined to estimate overall mean differences, and associated CIs were calculated using bootstrapping [13].

## Results

### Patient characteristics

**Fig. 1:**
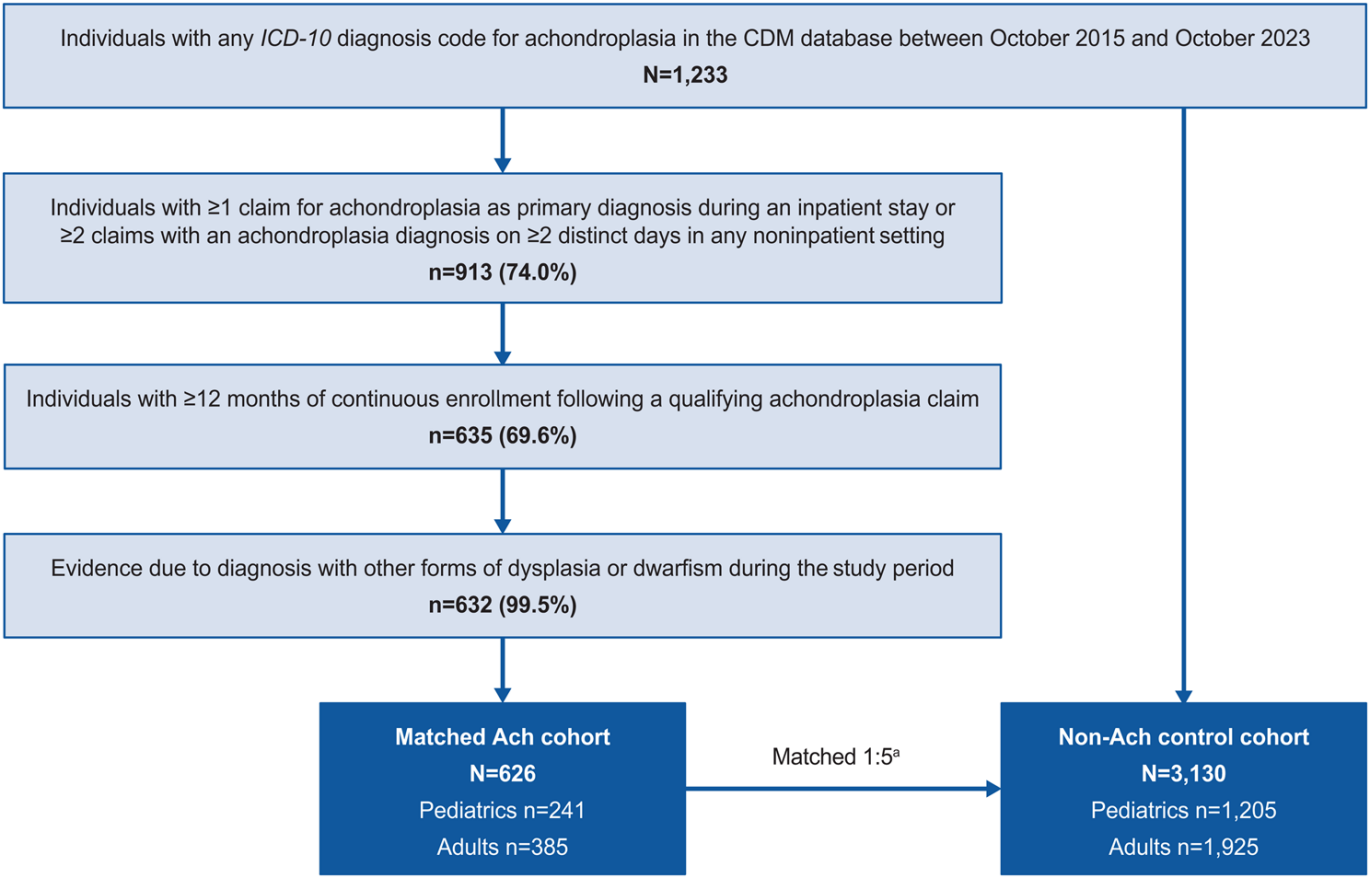
Cohort selection and attrition counts. Ach, achondroplasia; CDM, Optum’s deidentified Clinformatics^®^ Data Mart; *ICD*,* International Classification of Diseases.* ^a^People living with achondroplasia were matched to 5 people without achondroplasia based on age at index, sex, index date, and length of observation period; matched individuals had to be observable for at least the same number of days as the achondroplasia case

Overall, 626 people living with achondroplasia were matched with 3,130 people in the control cohort (Fig. 1). Mean (SD) age overall was 33.2 (24.4) years for people living with achondroplasia and 33.1 (24.3) years for the matched nonachondroplasia controls; both cohorts were 56.2% female. Subgroups of 241 pediatric and 385 adults with achondroplasia were matched with 1,205 and 1,925 nonachondroplasia controls, respectively. In children, mean (SD) age was 7.9 (5.4) years for achondroplasia and nonachondroplasia controls, 53.1% were female, and 54.4% of children with achondroplasia and 48.2% of matched nonachondroplasia controls were white (Table 1). In adults, mean age (SD) was 49.0 (17.3) years and 48.9 (17.1) years for achondroplasia and nonachondroplasia controls, respectively, and 58.2% in both groups were female. The majority of adults with achondroplasia and matched nonachondroplasia controls were White (68.8% vs. 68.5%).

**Table 1:** Patient characteristics at index date stratified by age and presence of spinal stenosis

	Pediatrics			Adults			Ach-SpS		
	Ach (*n* = 241)	Control (*n* = 1,205)^a^	*P* ^b^	Ach (*n* = 385)	Control (*n* = 1,925)^a^	*P* ^b^	Ach-SpS (*n* = 199)	Control (*n* = 995)^a^	*P* ^b^
**Mean age**,** (SD)**,** years**	7.9 (5.4)	7.9 (5.4)	1.00	49.0 (17.3)	48.9 (17.1)	0.92	40.1 (24.7)	40.1 (24.6)	0.98
**Sex**			1.00			1.00			1.00
Female	128 (53.1)	640 (53.1)	-	224 (58.2)	1120 (58.2)	-	106 (53.3)	530 (53.3)	-
Male	113 (46.9)	565 (46.9)	-	161 (41.8)	805 (41.8)	-	93 (46.7)	465 (46.7)	-
**Race/ethnicity**			0.27			0.05			0.08
White	131 (54.4)	581 (48.2)	-	265 (68.8)	1,318 (68.5)	-	136 (68.3)	607 (61.0)	-
Black	10 (4.1)	58 (4.8)	-	40 (10.4)	206 (10.7)	-	10 (5.0)	93 (9.4)	-
Asian	10 (4.1)	48 (4.0)	-	11 (2.9)	92 (4.8)	-	7 (3.5)	46 (4.6)	-
Other	11 (4.6)	96 (8.0)	-	31 (8.1)	188 (9.8)	-	13 (6.5)	102 (10.3)	-
Missing/unknown	79 (32.8)	422 (35.0)	-	38 (9.9)	121 (6.3)	-	33 (16.6)	147 (14.8)	-
**Insurance type**			< 0.01			< 0.01			< 0.01
Commercial	241 (100.0)	1205 (100.0)	-	155 (40.3)	1392 (72.3)	-	98 (49.2)	754 (75.8)	-
Medicare	0 (0.0)	0 (0.0)	-	230 (59.7)	533 (27.7)	-	101 (50.8)	241 (24.2)	-
Medicaid	0 (0.0)	0 (0.0)	-	0 (0.0)	0 (0.0)	-	0 (0.0)	0 (0.0)	-
Missing/unknown	0 (0.0)	0 (0.0)	-	0 (0.0)	0 (0.0)	-	0 (0.0)	0 (0.0)	-
**Region**			0.14			0.23			0.44
Northeast	18 (7.5)	148 (12.3)	-	52 (13.5)	200 (10.4)	-	26 (13.1)	124 (12.5)	-
Midwest	63 (26.1)	335 (27.8)	-	104 (27.0)	553 (28.7)	-	58 (29.1)	266 (26.7)	-
South	95 (39.4)	445 (36.9)	-	159 (41.3)	799 (41.5)	-	82 (41.2)	390 (39.2)	-
West	65 (27.0)	273 (22.7)	-	70 (18.2)	361 (18.8)	-	33 (16.6)	206 (20.7)	-
Other/unknown	0 (0.0)	< 5 (< 0.03)	-	0 (0.0)	12 (0.6)	-	0 (0.0)	9 (0.9)	-
**Follow-up**,** mean (SD)**,** days**	1,222.9 (741.0)	1,262.4 (729.0)	0.45	1,231.5 (686.1)	1,270.3 (679.2)	0.31	1,294.6 (698.7)	1,332.7 (691.6)	0.48

Ach, achondroplasia; SpS, spinal stenosis. ^a^People living with achondroplasia were matched to 5 people without achondroplasia based on age at index, sex, index date, and length of observation period. ^b^Continuous variables were compared using *t* tests; categorical variables were compared using Pearson’s chi-squared tests

### Patient comorbidities

The proportions of people experiencing different types of comorbidities over all follow-up, stratified by age, are presented in Table 2. A significantly greater proportion of children living with achondroplasia had skeletal complications compared with matched nonachondroplasia controls (62.7% vs. 4.1%; *P* < 0.01). Among these, the most common skeletal complications observed in children with achondroplasia were leg deformities (32.4% vs. 0.7%; *P* < 0.01) and spinal canal stenosis (19.1% vs. 0.0%; *P* < 0.01). Other nonskeletal complications were also more frequently observed in children with achondroplasia than in matched nonachondroplasia controls, including otitis media (55.2% vs. 34.4%; *P* < 0.01), pain (51.9% vs. 41.7%; *P* < 0.01), and sleep apnea (39.4% vs. 4.1%; *P* < 0.01).

**Table 2:** Comorbidities during all follow-up^a^ stratified by age and presence of spinal stenosis

	Pediatrics^b^		Adults^b^		Ach-SpS^b^	
	Ach (*n* = 241)	Control (*n* = 1,205)	Ach (*n* = 385)	Control (*n* = 1,925)	Ach-SpS (*n* = 199)	Control (*n* = 995)
**Mean CCI score (SD)**	0.32 (0.67)	0.17 (0.47)*	1.68 (2.20)	1.16 (2.09)*	1.56 (2.10)	1.05 (2.06)*
**Skeletal complications**	151 (62.7)	49 (4.1)*	289 (75.1)	633 (32.9)*	199 (100.0)	282 (28.3)*
Leg deformity	78 (32.4)	8 (0.7)*	37 (9.6)	24 (1.2)*	37 (18.6)	11 (1.1)*
Spinal canal stenosis	46 (19.1)	0 (0.0)*	151 (39.2)	138 (7.2)*	167 (83.9)	68 (6.8)*
Kyphosis	45 (18.7)	< 5 (< 0.2)*	33 (8.6)	19 (1.0)*	51 (25.6)	13 (1.3)*
Scoliosis	45 (18.7)	29 (2.4)*	68 (17.7)	48 (2.5)*	60 (30.2)	26 (2.6)*
Craniocervical foramen magnum stenosis	44 (18.3)	< 5 (< 0.1)*	81 (21.0)	63 (3.3)*	125 (62.8)	27 (2.7)*
Arthritis/osteoarthritis	< 5 (< 0.2)	5 (0.4)	188 (48.8)	500 (26.0)*	87 (43.7)	213 (21.4)*
Osteoporosis	0 (0.0)	< 5 (< 0.2)	66 (17.1)	116 (6.0)*	28 (14.1)	51 (5.1)*
**Other complications**	222 (92.1)	913 (75.8)*	375 (97.4)	1,694 (88.0)*	196 (98.5)	865 (86.9)*
Pain	125 (51.9)	502 (41.7)*	330 (85.7)	1,316 (68.4)*	173 (86.9)	630 (63.3)*
Otitis media	133 (55.2)	414 (34.4)*	69 (17.9)	133 (6.9)*	62 (31.2)	168 (16.9)*
Sleep apnea	95 (39.4)	50 (4.1)*	121 (31.4)	278 (14.4)*	93 (46.7)	126 (12.7)*
Other dysplasia/dwarfism	83 (34.4)	115 (9.5)*	221 (57.4)	716 (37.2)*	118 (59.3)	336 (33.8)*
Hearing impairment/loss	76 (31.5)	44 (3.7)*	98 (25.5)	152 (7.9)*	67 (33.7)	71 (7.1)*
Obesity	17 (7.1)	44 (3.7)	233 (60.5)	606 (31.5)*	101 (50.8)	255 (25.6)*
Hypertension	15 (6.2)	15 (1.2)*	230 (59.7)	814 (42.3)*	105 (52.8)	359 (36.1)*
Hyperlipidemia	< 5 (< 0.8)	20 (1.7)	170 (44.2)	787 (40.9)	76 (38.2)	351 (35.3)
Spinal stenosis	54 (22.4)	< 5 (< 0.1)*	145 (37.7)	165 (8.6)*	199 (100.0)	76 (7.6)*
Anxiety	38 (15.8)	149 (12.4)	163 (42.3)	563 (29.2)*	80 (40.2)	232 (23.3)*
Depression	22 (9.1)	78 (6.5)	141 (36.6)	381 (19.8)*	72 (36.2)	153 (15.4)*

Ach, achondroplasia; CCI, Charlson Comorbidity Index; SpS, spinal stenosis

*P < 0.01 vs. Ach value ^a^Individuals had variable follow-up, from index until death, censoring, or loss to follow-up. ^b^Continuous variables were compared using *t* tests; categorical variables were compared using Pearson’s chi-squared tests

Adults living with achondroplasia had a significantly higher mean (SD) CCI score than matched nonachondroplasia controls (1.7 [2.2] vs. 1.2 [2.1]; *P* < 0.01) and a significantly greater proportion had skeletal complications (75.1% vs. 32.9%; *P* < 0.01). The most prevalent skeletal complications were arthritis/osteoarthritis (48.8% vs. 26.0%), SpS (39.2% vs. 7.2%), and craniocervical foramen magnus stenosis (21.0% vs. 3.3%; all *P* < 0.01), while the most common nonskeletal complications were pain (85.7% vs. 68.4%), obesity (60.5% vs. 31.5%), and hypertension (59.7% vs. 42.3%; all *P* < 0.01). Mental health complications were also significantly more prevalent in adults with achondroplasia, including anxiety (42.3% vs. 29.3%) and depression (36.6% vs. 19.8%; both *P* < 0.01).

### HCRU and costs

**Fig. 2:**
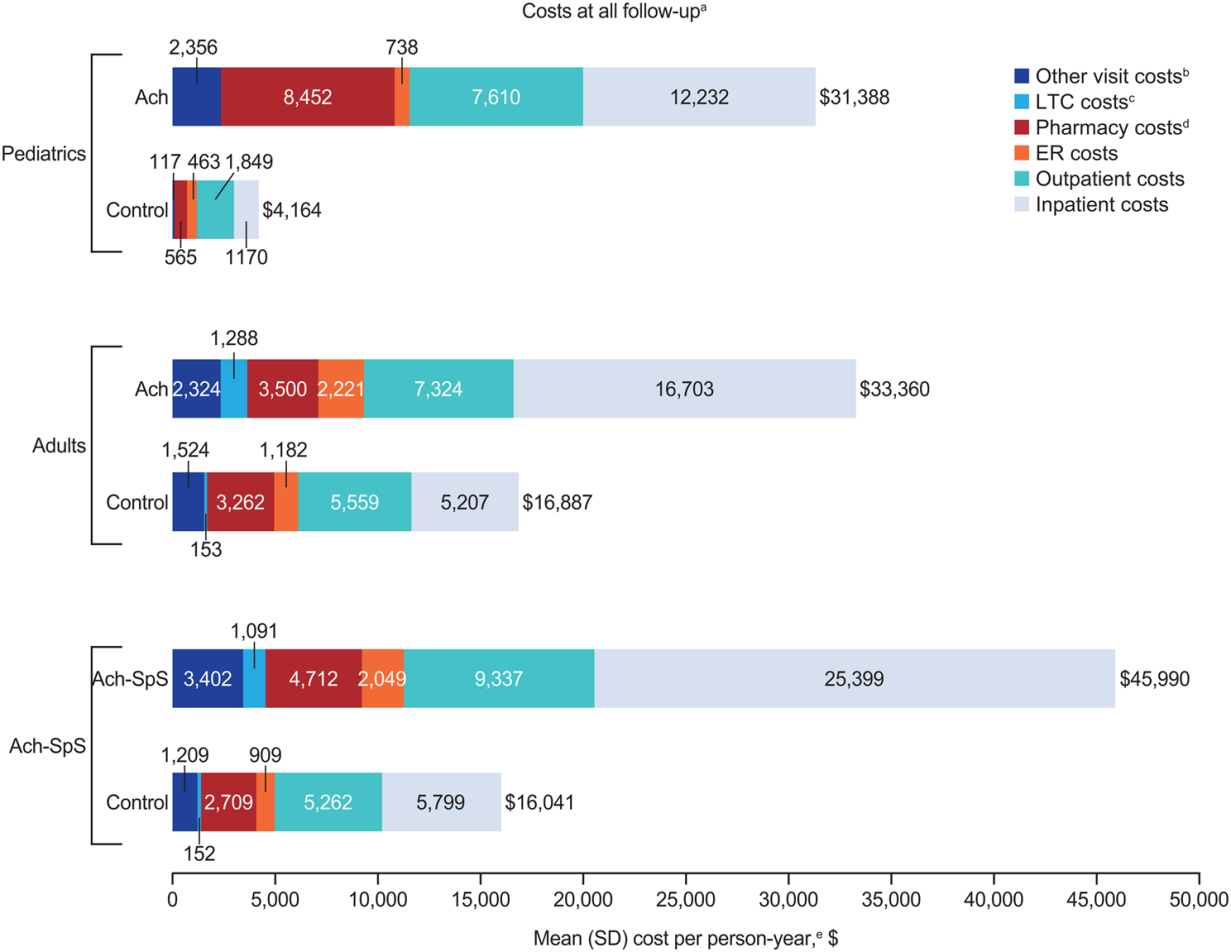
Healthcare costs during all follow-up^a^ stratified by age and presence of spinal stenosis. Ach, achondroplasia; ER, emergency room; LTC, long-term care; SpS, spinal stenosis. ^a^Individuals had variable follow-up, from index until death, censoring, or loss to follow-up. ^b^Other visits included complementary medicine services (e.g., physical medicine and rehabilitation procedures, physiotherapy, occupational therapy, chiropractic/osteopathic procedures, acupuncture, speech therapy, and medical imaging). ^c^Long-term care days included time spent in assisted living facilities, group homes, skilled nursing facilities, nursing facilities, and hospices; long-term care episodes could have overlapped with other episodes of care and were not mutually exclusive from care in inpatient, outpatient, ER, and other settings. ^d^Pharmacy costs were compared between groups using a 2-part model of logistic regression and gamma generalized linear model with identity link. ^e^Costs were compared using generalized linear models with a log link and robust variance. All tests compared people living with achondroplasia with matched nonachondroplasia controls

Annualized HCRU rates and associated healthcare costs over all available follow-up are shown in Table 3; Fig. 2, and Additional file: Table S4. Children living with achondroplasia were more likely to experience inpatient admissions (36.9% vs. 15.4%; *P* < 0.01) and other visits/complementary medicine services (65.1% vs. 42.3%; *P* < 0.01) than nonachondroplasia controls (Table 3). While the proportion of children with outpatient visits (100.0% vs. 99.3%; *P* = 0.37), ER visits (43.2% vs. 36.7%; *P* = 0.07), and pharmacy fills (88.0% vs. 85.7%; *P* = 0.41) were comparable between children with achondroplasia and nonachondroplasia controls, IRRs (95% CI) comparing the PPY rate of visits consistently indicated statistically significant higher HCRU incidence in children with achondroplasia across outpatient visits (13.18 vs. 6.28 PPY; IRR [95% CI]: 2.10 [2.05 to 2.15]), ER visits (0.30 vs. 0.20 PPY; IRR [95% CI]: 1.47 [1.27 to 1.69]), inpatient visits (0.30 vs. 0.09 PPY; IRR [95% CI]: 3.46 [2.94 to 4.08]), pharmacy fills (7.90 vs. 4.02 PPY; IRR [95% CI]: 1.97 [1.91 to 2.02]), and other visits (10.39 vs. 1.16 PPY; IRR [95% CI]: 8.94 [8.63 to 9.26]) (all *P* < 0.01).

**Table 3:** All-cause HCRU during all follow-up^a^ stratified by age and presence of spinal stenosis

	Pediatrics			Adults			Ach-SpS		
	Ach (*n* = 241)	Control (*n* = 1,205)	*P* ^b^	Ach (*n* = 385)	Control (*N* = 1,925)	*P* ^b^	Ach-SpS (*n* = 199)	Control (*n* = 995)	*P* ^b^
**Events**, ***n*** **(%)**									
Outpatient	241 (100.0)	1,196 (99.3)	0.37	383 (99.5)	1,883 (97.8)	0.05	198 (99.5)	979 (98.4)	0.38
ER	104 (43.2)	442 (36.7)	0.07	225 (58.4)	773 (40.2)	< 0.01	118 (59.3)	407 (40.9)	< 0.01
Inpatient	89 (36.9)	186 (15.4)	< 0.01	199 (51.7)	496 (25.8)	< 0.01	130 (65.3)	265 (26.6)	< 0.01
LTC^c^	0 (0.0)	0 (0.0)	NA	48 (12.5)	51 (2.6)	< 0.01	22 (11.1)	24 (2.4)	< 0.01
Pharmacy fills	212 (88.0)	1,033 (85.7)	0.42	370 (96.1)	1,778 (92.4)	0.01	192 (96.5)	913 (91.8)	0.03
Other^d^	157 (65.1)	510 (42.3)	< 0.01	345 (89.6)	1,455 (75.6)	< 0.01	181 (91.0)	699 (70.3)	< 0.01
**Incidence**,** PPY**									
Outpatient	13.18	6.28		13.88	10.04		17.47	9.65	
IRR (95% CI)	2.10 (2.05–2.15)		< 0.01	1.38 (1.36–1.41)		< 0.01	1.81 (1.77–1.85)		< 0.01
ER	0.30	0.20		0.63	0.36		0.61	0.34	
IRR (95% CI)	1.47 (1.27–1.69)		< 0.01	1.76 (1.63–1.91)		< 0.01	1.82 (1.63–2.03)		< 0.01
Inpatient	0.30	0.09		0.39	0.21		0.52	0.18	
IRR (95% CI)	3.46 (2.94–4.08)		< 0.01	1.88 (1.69–2.08)		< 0.01	2.83 (2.49–3.21)		< 0.01
LTC^c^	0.00	0.00		1.93	0.31		1.85	0.17	
IRR (95% CI)	NA		NA	6.31 (5.95–6.68)		< 0.01	10.81 (9.82–11.89)		< 0.01
Pharmacy fills	7.90	4.02		32.76	20.41		28.93	16.90	
IRR (95% CI)	1.97 (1.91–2.02)		< 0.01	1.61 (1.59–1.62)		< 0.01	1.71 (1.68–1.74)		< 0.01
Other^d^	10.39	1.16		14.06	5.55		18.24	4.84	
IRR (95% CI)	8.94 (8.63–9.26)		< 0.01	2.53 (2.49–2.58)		< 0.01	3.77 (3.68–3.85)		< 0.01

Ach, achondroplasia; ER, emergency room; HCRU, healthcare resource utilization; IRR, incidence rate ratios; LTC, long-term care; NA, not applicable; PPY, per person-year; SpS, spinal stenosis

^a^Individuals had variable follow-up, from index until death, censoring, or loss to follow-up

^b^HCRU was compared using generalized linear models with a log link and Poisson distribution. All tests compared people living with achondroplasia to matched nonachondroplasia controls

^c^Long-term care days included time spent in assisted living facilities, group homes, skilled nursing facilities, nursing facilities, and hospices; long-term care episodes could have overlapped with other episodes of care and were not mutually exclusive from care in inpatient, outpatient, ER, and other settings

^d^Other visits included complementary medicine services (e.g., physical medicine and rehabilitation procedures, physiotherapy, occupational therapy, chiropractic/osteopathic procedures, acupuncture, speech therapy, and medical imaging)

Mean annualized total healthcare costs were significantly higher in children with achondroplasia ($31,388 vs. $4,164 PPY; mean difference [95% CI]: $27,224 [$20,070 to $35,671]; *P* < 0.01) compared with matched nonachondroplasia controls (Fig. 2; Additional file: Table S4). The difference in total annualized healthcare costs appeared to be predominantly driven by the large differences in inpatient costs ($12,232 vs. $1,170 PPY; mean difference [95% CI]: $11,062 [$6,823-$16,383]; *P* < 0.01), pharmacy costs ($8,452 vs. $565 PPY; mean difference [95% CI]: $7,887 [$3,876-$12,672]; *P* < 0.01), and outpatient costs ($7,610 PPY vs. $1,849 PPY; mean difference [95% CI]: $5,761 [$4,480-$7,396]; *P* < 0.01).

The proportion of adults living with achondroplasia with outpatient visits (99.5% vs. 97.8%; *P* = 0.05) and pharmacy fills (96.1% vs. 92.4%; *P* = 0.01) was slightly but significantly higher than nonachondroplasia controls (Table 3); adults with achondroplasia were also significantly more likely to experience all-cause ER visits (58.4% vs. 40.2%), inpatient admissions (51.7% vs. 25.8%), LTC stays (12.5% vs. 2.7%), and other visits (89.6% vs. 75.6%) (all *P* < 0.01), compared with nonachondroplasia controls. Comparison of incidence rates for outpatient visits (13.88 vs. 10.04 PPY; IRR [95% CI]: 1.38 [1.36–1.41]), inpatient admissions (0.39 vs. 0.21 PPY; IRR [95% CI]: 1.88 [1.69–2.08]), LTC (1.93 vs. 0.31 PPY; IRR [95% CI]: 6.31 [5.95–6.68]), and other visits (14.06 vs. 5.55 PPY; IRR [95% CI]: 2.53 [2.49–2.58]) indicated a greater HCRU burden in adults with achondroplasia than nonachondroplasia controls (all *P* < 0.01).

Mean annualized total healthcare costs were significantly higher in adults with achondroplasia ($33,360 vs. $16,887 PPY; mean difference [95% CI]: $16,472 [$11,260-$21,885]; *P* < 0.01) compared with matched nonachondroplasia controls (Fig. 2; Additional file: Table S4). The difference in total healthcare costs was predominantly driven by the large differences in inpatient admission costs ($16,703 vs. $5,207 PPY, mean difference [95% CI]: $11,496 [$7,910-$15,488]; *P* < 0.01) and outpatient visit costs ($7,324 PPY vs. $5,559 PPY, mean difference [95% CI]: $1,765 [$498-$3,170]; *P* = 0.01).

### Spinal stenosis subgroup analyses

A subgroup analysis assessed clinical outcomes in people living with Ach-SpS (*n* = 199; 74.6% >18 years at index date). Skeletal complications were substantially more frequent in the subgroup of people living with Ach-SpS (100.0%) compared with pediatric (62.7%) and adult (75.1%) achondroplasia cohorts (Table 2). The most common skeletal complications in people living with Ach-SpS included spinal canal stenosis (83.9% vs. 19.1% and 39.2% in children and adults with achondroplasia, respectively) and craniocervical foramen magnus stenosis (62.8% vs. 18.3% and 21.0% in children and adults with achondroplasia, respectively). People living with Ach-SpS commonly reported also nonskeletal complications, most frequently pain (86.9% vs. 51.9% and 85.7% in children and adults with achondroplasia, respectively) and other dysplasia or dwarfism (59.3% vs. 34.4% and 57.4% in children and adults with achondroplasia, respectively). While people living with Ach-SpS had comparable prevalences of anxiety and depression compared with adults with achondroplasia, these comorbidities were numerically higher in this group compared with children with achondroplasia (anxiety: 40.2%, vs. 15.8% and 42.3%; depression: 36.2%, vs. 9.1% and 36.6% in children and adults with achondroplasia, respectively).

People living with Ach-SpS frequently experienced inpatient admissions (65.3% vs. 36.9% and 51.7% in children and adults with achondroplasia, respectively). PPY incidence was higher in people living with Ach-SpS subgroup than in children and adults with achondroplasia for outpatient visits (17.47 PPY vs. 13.18 PPY and 13.88 PPY in children and adults with achondroplasia, respectively) and inpatient admissions (0.52 PPY vs. 0.30 PPY and 0.39 PPY in children and adults with achondroplasia, respectively) (Table 3). People living with Ach-SpS incurred substantial annualized total healthcare costs ($45,990 vs. $31,388 and $33,360 in children and adults with achondroplasia, respectively), largely driven by inpatient costs ($25,399 vs. $12,232 and $16,703 in children and adults with achondroplasia, respectively) (Fig. 2; Additional file: Table S4).

## Discussion

Individuals living with achondroplasia experience substantial clinical and economic burden of disease, with life-long complications arising from early childhood. Current standard of care for achondroplasia often includes regular monitoring of growth and development, screening for respiratory and hearing problems, neurological evaluations, and adoption of mobility devices to maximize independence during childhood [14]. While standard interventions involve treatment with growth hormone therapy, its effectiveness remains unclear [14, 15]. As such, standard management and treatment pathways are fragmented and often costly due to the need for coordinated and proactive care across specialist areas. This retrospective database study demonstrated an increased prevalence of comorbidities children and adults living with achondroplasia compared with nonachondroplasia controls in multiple domains, including otorhinolaryngology, musculoskeletal, cardiovascular, and pain; in addition, an increase in HCRU associated with the higher prevalences of comorbidities was observed coupled with markedly higher costs in the achondroplasia cohort compared with nonachondroplasia controls. These observations emphasize the multisystemic burden of achondroplasia in current clinical practice and may further inform the ongoing clinical development of disease-modifying therapies.

Although increased comorbidity burden, HCRU, and costs have been described in prior studies [16–21], we extend previous findings by comparing comorbidities in people living with achondroplasia and nonachondroplasia controls and assessing the commensurate increase in both HCRU and HCRU-related costs. The HCRU burden in both children and adults with achondroplasia was considerable, with people frequently experiencing ER visits (43–58%) and/or inpatient admissions (37–52%) during follow-up. In fact, people living with achondroplasia were 1.4 to 3.5 times more likely to have outpatient visits, ER visits, inpatient admissions, and pharmacy fills than nonachondroplasia controls. This increase in HCRU was also evident in the higher HCRU-related costs incurred in the achondroplasia cohort, where inpatient admissions and pharmacy fills accounted for around 66% of the higher total costs observed in the pediatric achondroplasia cohort, and outpatient and inpatient admissions accounted for 72% of the higher total cost in adults with achondroplasia. These findings are consistent with prior available studies. A UK real-world cohort study reported significantly higher HCRU rates in people living with achondroplasia compared with matched people without achondroplasia, with higher rates of general practitioner visits, outpatient admissions, inpatient admissions, and medical imaging [19]. Children and adults from the US National Inpatient sample also incurred higher mean total inpatient costs than the national average (children, $22,031 vs. $7,935, respectively; adults, $18,224 vs. $12,931, respectively) [20]. In one recent claims database study which also assessed economic burden of achondroplasia in children and adults (2008 to 2021), Merchant et al. reported HCRU and costs comparable with the present study, with around a 2-fold increase in outpatient visits and 1.5-fold increase in prescriptions, and higher total healthcare costs in the achondroplasia cohort (children, $20,336 vs. $73; adults, $21,579 vs. $4,951) [21]. Although the total costs reported in the present study were slightly higher than in the Merchant study, this is likely attributed to several factors, including the long-term impacts of the COVID-19 pandemic on healthcare resource utilization and the approval of vosoritide in 2021 which may have impacted patterns of healthcare seeking and disease monitoring. Additionally, inclusion criteria in this study were more restrictive than those in the prior claims database study, which may have resulted in identification of individuals with more severe achondroplasia incurring higher costs. Nonetheless, our study findings remain relevant and provide up-to-date information on current clinical and economic burden of achondroplasia in the present healthcare landscape. These data collectively highlight that across a range of ages, people living with achondroplasia incur higher costs associated with HCRU compared with people without achondroplasia.

These observations also highlight the multisystem impact of achondroplasia beyond the skeletal system, with higher rates of nonskeletal conditions, such as sleep apnea and hypertension, as well as associated mental health consequences, such as anxiety and depression. A high prevalence of sleep apnea was observed in both children and adults with achondroplasia, in accordance with prior studies [22]. Although the CDM database in this study does not provide information about the type of sleep disordered breathing, prior studies have documented that both central and obstructive sleep apnea may be present both within and across individuals. Potential mechanisms for sleep apnea in patients with achondroplasia include the effects of increased body weight on upper airway collapsibility, bony and soft tissue distortion of the upper airway, and foramen magnum stenosis with potential impairment of central ventilatory drive [23]. However, the exact contribution of these factors likely varies across individuals, potentially impacting the optimal therapeutic approach. While the prevalence of hyperlipidemia was similar between people living with achondroplasia and people without achondroplasia, significant increases in the prevalence of obesity and hypertension were noted in people living with achondroplasia. However, this study could not identify the presence of an associated metabolic syndrome and further research on the prevalence of metabolic syndrome will be beneficial.

This study also provides valuable information on the higher burden of people living with Ach-SpS; these individuals had the highest prevalence of spinal canal stenosis, pain, and other dysplasia or dwarfism comorbidities and incurred the greatest HCRU and costs. A prior real-world study demonstrated significantly poorer patient-reported outcomes in Norwegian people living with Ach-SpS compared with those who did not have SpS, in which people with Ach-SpS reported increased pain, worse physical functioning, and more limitations in daily activities [24]. However, more research is needed to further identify additional subgroups of people living with achondroplasia with especially high burden, which may help improve management of the disease. While disease-modifying therapy was recently approved, there were insufficient data to evaluate its impact on the observed comorbidities and HCRU.

As with any real-world analysis, these analyses were limited by the potential for missing or incomplete records and coding errors. Another limitation is that only services billed for and diagnoses that have an associated *ICD-10* code are reflected in the administrative claims data; thus, some clinical comorbidities and symptoms, such as pain, may be underestimated. The identification of individuals using *ICD-10* codes may also be considered a limitation, as there is a potential for misidentification of individuals with hypochondroplasia and osteosclerosis congenita using the code Q77.4. However, as osteosclerosis congenita and hypochondroplasia have low prevalences [25, 26], and hypochondroplasia presents with milder clinical manifestations, inclusion of individuals with these conditions may have resulted in conservative estimates of disease burden for achondroplasia. As Optum applies standard pricing algorithms to claims in the database to account for differences in pricing across health plans and provider contracts, cost figures were adjusted estimates and may not be the true amounts paid by the payer. Moreover, HCRU and costs may be underestimated in the study, as denied claims (which may represent services that are provided to the member but not paid by the insurer), services and medical devices/equipment not covered by insurance, and costs of over-the-counter medications may not be captured in the Optum CDM. While direct matching was performed by age at index date, sex, calendar date of index date and observation period duration, other clinical differences were not adjusted for in the comparative analyses; as regression analysis adjustment is only necessary in variable ratio matching to address confounding bias, this is not the case for fixed ratio matching which was used in this study. Additionally, as achondroplasia is an inborn disease with multiple manifestations, other clinical differences observed between cohorts were assumed to be driven by achondroplasia manifestations and were therefore not adjusted for in the comparative analyses. As claims databases do not provide information on reasons for treatment decision-making, future analyses using electronic medical record-linked modelling, including narrative notes and chart entries, are warranted to evaluate achondroplasia care standards. Finally, the results of this study may not be fully generalizable to non-US populations and individuals without coverage from a commercial health and/or Medicare Advantage, as people who are uninsured or who are covered by other insurance schemes such as Medicaid and the Children’s Health Insurance Program may not have been captured in this study. Therefore, the findings of this study may not be representative of these populations and further analyses assessing these underrepresented patients are warranted.

In this real-world US retrospective cohort study, children and adults with achondroplasia had significantly higher clinical and HCRU burden compared with matched nonachondroplasia controls; a subset of people living with Ach-SpS incurred even greater HCRU and costs. These data emphasize the unmet medical need for more effective disease-modifying therapies by describing the clinical and healthcare resource burden associated with achondroplasia.

## Supplementary Information

Below is the link to the electronic supplementary material.

Supplementary Material 1

## Data Availability

The dataset supporting the conclusions of this article is included within the article and its additional files.
