# Supplementary material for: Clinical burden and healthcare resource utilization associated with achondroplasia: a real-world observational, retrospective cohort study
Source: Orphanet J Rare Dis. 2025 Nov 5;20:563. doi: 10.1186/s13023-025-04072-w (PMC12587645; doi:10.1186/s13023-025-04072-w)
Supplement: Supplementary file 1 — Supplementary Material 1 [file 13023_2025_4072_MOESM1_ESM.docx]

**Additional file. Table S1. Comorbidities during 12-month follow-up stratified by age and presence of spinal stenosis**

|  | **Pediatrics** | | **Adults** | | **Ach-SpS cohort** | |
| --- | --- | --- | --- | --- | --- | --- |
|  | **Ach**  **(n=241)** | **Control**  **(n=1,205)** | **Ach**  **(n=385)** | **Control**  **(n=1,925)** | **Ach-SpS**  **(n=138)** | **Control**  **(n=690)** |
| **Mean CCI score (SD)** | 0.18 (0.51) | 0.10 (0.32) | 1.05 (1.53) | 0.63 (1.36)* | 0.94 (1.42) | 0.57 (1.29) |
| **Skeletal complications** | 118 (49.0) | 23 (1.9)* | 237 (61.6) | 353 (18.3)* | 137 (99.3) | 110 (15.9)* |
| Leg deformity | 50 (20.7) | <5 (<0.2)* | 20 (5.2) | 5 (0.3)* | 9 (6.5) | <5 (<0.3)* |
| Spinal canal stenosis | 32 (13.3) | 0 (0.0)* | 111 (28.8) | 52 (2.7)* | 111 (80.4) | 19 (2.8)* |
| Kyphosis | 32 (13.3) | <5 (<0.1)* | 23 (6.0) | 10 (0.5)* | 31 (22.5) | <5 (<0.4)* |
| Scoliosis | 23 (9.5) | 15 (1.2)* | 51 (13.2) | 25 (1.3)* | 33 (23.9) | 10 (1.4)* |
| Craniocervical foramen   magnum stenosis | 31 (12.9) | <5 (<0.4)* | 51 (13.2) | 18 (0.9)* | 82 (59.4) | 6 (0.9)* |
| Arthritis/osteoarthritis | <5 (<2) | <5 (<0.1) | 125 (32.5) | 268 (13.9)* | 34 (24.6) | 77 (11.2)* |
| Osteoporosis | 0 (0.0) | <5 (<0.1) | 47 (12.2) | 60 (3.1)* | 11 (8.0) | 19 (2.8)* |
| **Other complications** | 199 (82.6) | 688 (57.1)* | 355 (92.2) | 1,480 (76.9)* | 135 (97.8) | 510 (73.9)* |
| Pain | 77 (32.0) | 250 (20.8)* | 267 (69.4) | 886 (46.0)* | 108 (78.3) | 281 (40.7)* |
| Otitis media | 103 (42.7) | 275 (22.8)* | 36 (9.4) | 50 (2.6)* | 27 (19.6) | 67 (9.7)* |
| Sleep apnea | 82 (34.0) | 20 (1.7)* | 90 (23.4) | 167 (8.7)* | 57 (41.3) | 58 (8.4)* |
| Other dysplasia/dwarfism | 61 (25.3) | 66 (5.5)* | 169 (43.9) | 458 (23.8)* | 68 (49.3) | 135 (19.6)* |
| Hearing impairment/loss | 45 (18.7) | 15 (1.2)* | 57 (14.8) | 67 (3.5)* | 28 (20.3) | 16 (2.3)* |
| Obesity | 8 (3.3) | 20 (1.7) | 158 (41.0) | 376 (19.5)* | 51 (37.0) | 115 (16.7)* |
| Hypertension | 9 (3.7) | 8 (0.7)* | 204 (53.0) | 652 (33.9)* | 58 (42.0) | 202 (29.3)* |
| Hyperlipidemia | <5 (<0.4) | 9 (0.7) | 136 (35.3) | 531 (27.6)* | 37 (26.8) | 158 (22.9)* |
| Spinal stenosis | 35 (14.5) | <5 (<0.1)* | 103 (26.8) | 65 (3.4)* | 138 (100) | 23 (3.3)* |
| Anxiety | 13 (5.4) | 62 (5.1) | 118 (30.6) | 341 (17.7)* | 40 (29.0) | 103 (14.9)* |
| Depression | 9 (3.7) | 34 (2.8) | 101 (26.2) | 245 (12.7)* | 32 (23.2) | 70 (10.1)* |

Ach, achondroplasia; CCI, Charlson Comorbidity Index; SpS, spinal stenosis.

**P*<0.01 vs Ach value.
^a^Continuous variables were compared using *t* tests; categorical variables were compared using Pearson’s chi-squared tests.

**Additional file. Table S2. All-cause HCRU during 12-month follow-up stratified by age and presence of spinal stenosis**

|  | **Pediatrics** | | | **Adults** | | | **Ach-SpS** | | |
| --- | --- | --- | --- | --- | --- | --- | --- | --- | --- |
|  | **Ach**  **(n=241)** | **Control**  **(n=1,205)** | ***P*^a^** | **Ach**  **(n=385)** | **Control**  **(N=1,925)** | ***P*^a^** | **Ach-SpS**  **(n=138)** | **Control**  **(n=690)** | ***P*^a^** |
| **Events, n (%)** | | | | | | | | | |
| Outpatient | 241 (100.0) | 1,186 (98.4) | 0.01 | 377 (97.9) | 1,830 (95.1) | 0.02 | 135 (97.8) | 664 (96.2) | 0.50 |
| ER | 58 (24.1) | 247 (20.5) | 0.25 | 131 (34.0) | 391 (20.3) | <0.01 | 38 (27.5) | 142 (20.6) | 0.09 |
| Inpatient | 62 (25.7) | 132 (11.0) | <0.01 | 137 (35.6) | 214 (11.1) | <0.01 | 69 (50.0) | 108 (15.7) | <0.01 |
| LTC^b^ | 0 (0.0) | 0 (0.0) | NA | 28 (7.3) | 18 (0.9) | <0.01 | 10 (7.2) | 9 (1.3) | <0.01 |
| Pharmacy fills | 184 (76.4) | 828 (68.7) | 0.02 | 358 (93.0) | 1,631 (84.7) | <0.01 | 127 (92.0) | 561 (81.3) | <0.01 |
| Other^c^ | 111 (46.1) | 263 (21.8) | <0.01 | 287 (74.6) | 1,010 (52.5) | <0.01 | 106 (76.8) | 315 (45.7) | <0.01 |
| **Incidence, PPY** | | | | | | | | | |
| Outpatient | 15.01 | 7.59 |  | 15.66 | 10.10 |  | 20.76 | 10.48 |  |
| IRR (95% CI) | 1.98 (1.90-2.06) | | <0.01 | 1.55 (1.51-1.60) | | <0.01 | 1.98 (1.90-2.07) | | <0.01 |
| ER | 0.38 | 0.28 |  | 0.75 | 0.35 |  | 0.60 | 0.32 |  |
| IRR (95% CI) | 1.37 (1.09-1.72) | | <0.01 | 2.13 (1.86-2.45) | | <0.01 | 1.89 (1.47-2.43) | | <0.01 |
| Inpatient | 0.46 | 0.14 |  | 0.56 | 0.25 |  | 0.82 | 0.32 |  |
| IRR (95% CI) | 3.25 (2.56-4.14) | | <0.01 | 2.23 (1.90-2.62) | | <0.01 | 2.57 (2.05-3.22) | | <0.01 |
| LTC^b^ | 0.00 | 0.00 |  | 3.08 | 0.65 |  | 3.31 | 0.30 |  |
| IRR (95% CI) | NA | | NA | 4.73 (4.37-5.12) | | <0.01 | 10.85 (9.22-12.78) | | <0.01 |
| Pharmacy fills | 7.73 | 3.79 |  | 32.48 | 19.57 |  | 27.18 | 15.96 |  |
| IRR (95% CI) | 2.04 (1.93-2.15) | | <0.01 | 1.66 (1.63-1.69) | | <0.01 | 1.70 (1.64-1.77) | | <0.01 |
| Other^c^ | 11.92 | 1.19 |  | 14.77 | 5.33 |  | 21.23 | 5.46 |  |
| IRR (95% CI) | 9.98 (9.37-10.63) | | <0.01 | 2.77 (2.68-2.86) | | <0.01 | 3.89 (3.71-4.08) | | <0.01 |

Ach, achondroplasia; ER, emergency room; HCRU, healthcare resource utilization; IRR, incidence rate ratio; LTC, long-term care; NA, not applicable; PPY, per person-year; SpS, spinal stenosis.

^a^HCRU was compared using generalized linear models with a log link and Poisson distribution. All tests compared people living with achondroplasia to matched nonachondroplasia controls.

^b^Long-term care days included time spent in assisted living facilities, group homes, skilled nursing facilities, nursing facilities, and hospices; long-term care episodes could have overlapped with other episodes of care and were not mutually exclusive from care in inpatient, outpatient, ER, and other settings.

^c^Other visits included complementary medicine services (eg, physical medicine and rehabilitation procedures, physiotherapy, occupational therapy, chiropractic/osteopathic procedures, acupuncture, speech therapy, and medical imaging).

**Additional file. Table S3. Healthcare costs during 12-month follow-up stratified by age and presence of spinal stenosis**

|  | **Pediatrics** | | **Adults** | | **Ach-SpS** | |
| --- | --- | --- | --- | --- | --- | --- |
|  | **Ach**  **(n=241)** | **Control**  **(n=1,205)** | **Ach**  **(n=385)** | **Control**  **(n=1,925)** | **Ach-SpS**  **(n=138)** | **Control**  **(n=690)** |
| **Costs, (SD), $** | | | | | | |
| Total healthcare costs | 32,202 (59,932) | 5,482 (22,436) | 42,008 (74,270) | 16,820 (58,875) | 72,637 (104,819) | 16,995 (75,021) |
| MD (95% CI)^a^ | 26,720 (19,410-34,605); <0.01 | | 25,118 (17,562-33,314); <0.01 | | 55,642 (37,940-74,389); <0.01 | |
| Outpatient costs | 9,021 (11,650) | 2,213 (4,655) | 8,072 (13,091) | 5,420 (14,390) | 11,810 (13,216) | 4,997 (13,001) |
| MD (95% CI)^a^ | 6,808 (5,417- 8,301); <0.01 | | 2,652 (1,284-4,199); <0.01 | | 6,812 (4,501-9,367); <0.01 | |
| ER costs | 799 (2,213) | 552 (1,693) | 2,570 (5,881) | 1,278 (9,644) | 1,996 (4,823) | 998 (3,354) |
| MD (95% CI)^a^ | 247 (32- 548); 0.09 | | 1,292 (538-2,027); <0.01 | | 998 (171- 1,869); 0.02 | |
| Inpatient costs | 15,489 (46,939) | 2,092 (19,650) | 23,572 (64,184) | 5,389 (45,449) | 49,252 (97,182) | 7,253 (70,647) |
| MD (95% CI)^a^ | 13,397 (7,738-19,689); <0.01 | | 18,183 (11,688-25,073); <0.01 | | 41,999 (25,937-60,155); <0.01 | |
| LTC^b^ costs | 0 (0) | 0 (0) | 1,839 (9,922) | 169 (2,511) | 2,318 (13,152) | 160 (1,575) |
| MD (95% CI)^a^ | NA | | 1,670 (796-2,794); <0.01 | | 2,158 (432-4,831); 0.06 | |
| Pharmacy costs^c^ | 3,965 (27,336) | 501 (3,659) | 3,269 (14,707) | 2,981 (14,004) | 3,006 (10,479) | 2,257 (9,330) |
| MD (95% CI)^a^ | 3,465 (547-7,194); 0.05 | | 288 (-1,053 to 2,013); 0.71 | | 749 (-854 to 2,739); 0.42 | |
| Other visit costs^d^ | 2,928 (13,652) | 124 (785) | 2,687 (6,102) | 1,583 (17,017) | 4,256 (8,978) | 1,329 (9,380) |
| MD (95% CI)^a^ | 2,803 (1,319-4,792); <0.01 | | 1,104 (120-2,000); 0.02 | | 2,927 (1,379-4,658); <0.01 | |

Ach, achondroplasia; SpS, spinal stenosis; ER, emergency room; LTC, long-term care; NA, not applicable; MD, mean difference.

**P*<0.05 vs Ach value.

^a^95% CIs and *P* values for costs were calculated using a logistic regression model. All tests compared people living with achondroplasia with matched nonachondroplasia using generalized linear models with a log link and robust variance estimator to account for matching in model 2 of the 2-part model.

^b^Long-term care days included time spent in assisted living facilities, group homes, skilled nursing facilities, nursing facilities, and hospices; long-term care episodes can overlap with other episodes of care and are not mutually exclusive from care in inpatient, outpatient, ER, and other settings.

^c^Pharmacy costs were compared between groups using a 2-part model of logistic regression and gamma generalized linear model with identity link.

^d^Other visits included complementary medicine services (eg, physical medicine and rehabilitation procedures, physiotherapy, occupational therapy, chiropractic/osteopathic procedures, acupuncture, speech therapy, and medical imaging).

**Additional file. Table S4. Healthcare costs during all follow-up^a^ stratified by age and presence of spinal stenosis**

|  | **Pediatrics** | | **Adults** | | **Ach-SpS** | |
| --- | --- | --- | --- | --- | --- | --- |
|  | **Ach**  **(n=241)** | **Control**  **(n=1,205)** | **Ach**  **(n=385)** | **Control**  **(n=1,925)** | **Ach-SpS**  **(n=199)** | **Control**  **(n=995)** |
| **Costs, (SD), $** | | | | | | |
| Total healthcare costs | 31,388 (59,435) | 4,164 (12,092) | 33,360 (47,878) | 16,887 (45,954) | 45,990 (53,517) | 16,041 (46,830) |
| MD (95% CI)^a^ | 27,224 (20,070-35,671); <0.01 | | 16,472 (11,260-21,885); <0.01 | | 29,949 (22,281-38,319); <0.01 | |
| Outpatient costs | 7,610 (11,658) | 1,849 (3,298) | 7,324 (11,696) | 5,559 (14,482) | 9,337 (9,085) | 5,262 (15,474) |
| MD (95% CI)^a^ | 5,761 (4,480-7,396); <0.01 | | 1,765 (498-3,170); 0.01 | | 4,074 (2,418-5,615); <0.01 | |
| ER costs | 738 (1,821) | 463 (1,154) | 2,220 (4,070) | 1,182 (4,375) | 2,049 (3,211) | 909 (2,457) |
| MD (95% CI)^a^ | 275 (57-520); 0.02 | | 1,039 (616-1,500); <0.01 | | 1,140 (658–1,633); <0.01 | |
| Inpatient costs | 12,232 (37,172) | 1,170 (9,192) | 16,703 (36,767) | 5,207 (28,934) | 25,399 (44,729) | 5,799 (36,680) |
| MD (95% CI)^a^ | 11,062 (6,823-16,383); <0.01 | | 11,496 (7,910-15,488); <0.01 | | 19,599 (13,365-26,443); <0.01 | |
| LTC^b^ costs | 0 (0) | 0 (0) | 1,288 (5,602) | 153 (1,523) | 1,091 (4,312) | 152 (1,334) |
| MD (95% CI)^a^ | NA | | 1,134 (627-1,748); <0.01 | | 939 (367-1,636); <0.01 | |
| Pharmacy costs^c^ | 8,452 (35,722) | 565 (3,804) | 3,500 (14,665) | 3,262 (14,451) | 4,172 (19,064) | 2,709 (12,178) |
| MD (95% CI)^a^ | 7,887 (3,876-12,672)^e^; <0.01^e^ | | 238 (-1,154 to 2,135); 0.79 | | 2,003 (-360 to 5,076); 0.15 | |
| Other visit costs^d^ | 2,356 (9,642) | 117 (669) | 2,324 (5,030) | 1,524 (13,076) | 3,402 (9,113) | 1,209 (8,417) |
| MD (95% CI)^a^ | 2,239 (1,127-3,636); <0.01 | | 800 (3-1,551); 0.04 | | 2,194 (961-3,656); <0.01 | |

Ach, achondroplasia; SpS, spinal stenosis; ER, emergency room; LTC, long-term care; NA, not applicable; MD, mean difference.

**P*<0.05 vs Ach value.

^a^95% CIs and *P* values for costs were calculated using a logistic regression model. All tests compared people living with achondroplasia with matched nonachondroplasia using generalized linear models with a log link and robust variance estimator.

^b^Long-term care days included time spent in assisted living facilities, group homes, skilled nursing facilities, nursing facilities, and hospices; long-term care episodes can overlap with other episodes of care and are not mutually exclusive from care in inpatient, outpatient, ER, and other settings.

^c^Pharmacy costs were compared between groups using a 2-part model of logistic regression and gamma generalized linear model with identity link.

^d^Other visits included complementary medicine services (eg, physical medicine and rehabilitation procedures, physiotherapy, occupational therapy, chiropractic/osteopathic procedures, acupuncture, speech therapy, and medical imaging).
